# Effects of Fe^3+^ on Acute Toxicity and Regeneration of Planarian (*Dugesia japonica*) at Different Temperatures

**DOI:** 10.1155/2019/8591631

**Published:** 2019-08-22

**Authors:** Xue Ding, Linxia Song, Yahong Han, Yingbo Wang, Xiaowang Tang, Guicai Cui, Zhenbiao Xu

**Affiliations:** College of Life Sciences, Shandong University of Technology, Zibo 255049, China

## Abstract

**Objective:**

To investigate the effects of different concentrations of Fe^3+^ on the acute toxicity and regeneration of planarian at different temperatures.

**Method:**

The planarians were treated with 40 mg/l, 50 mg/l, 60 mg/l, and 70 mg/l Fe^3+^ solution and placed in 15°C, 20°C, and 25°C, respectively, to observe the mortality and the poisoning pattern of the planarian. In addition, the planarians were cut into three parts of head, trunk, and tail, then placed in Fe^3+^ solution at concentrations of 10 mg/l, 15 mg/l, 20 mg/l, and 30 mg/l, and placed in 15°C, 20°C, and 25°C respectively, and the regeneration rate of the planarian was investigated.

**Results:**

At the same temperature, in the concentration of Fe^3+^ from 40 mg/l to 70 mg/l, the mortality of the planarian increased with the increasing of the concentration of Fe^3+^; at the same concentration and different temperatures, the death speed of the planarian is the fastest at 20°C, the next at 25°C, and the lowest at 15°C, indicating that the toxic effect of Fe^3+^ can be accelerated at a suitable temperature of 20°C. At the same temperature, in the low concentration of Fe^3+^ from 10 mg/l to 30 mg/l, the regeneration rate of the planarian gradually decreased with the increasing of the concentration of Fe^3+^; at the same concentration and different temperature, the regeneration rate of planarian was faster at 20°C and 25°C, but the difference between 20°C and 25°C was small, and the slowest at 15°C, indicating that the low temperature significantly affects the planarian regeneration speed. The study also found the regeneration rates of the head, trunk, and tail of the planarian were different; the head regeneration was the fastest, the trunk was the second, and the tail was the slowest.

**Conclusion:**

Fe^3+^ had obvious toxic effects on the survival and regeneration of planarian; the planarian is sensitive to Fe^3+^ and may be used to detect Fe^3+^ water pollution; in addition, temperature can affect the toxic effects of Fe^3+^ and thus affect the survival and regeneration of the planarian. Therefore, the temperature should be taken into consideration when detecting water Fe^3+^ pollution.

## 1. Introduction

In recent years, people have paid more and more attention to environmental issues, and the development of modern industry and agriculture has caused a large number of heavy metal ions to accumulate continuously in the water, causing serious harm to the water environment [1, 2].

Iron is an indispensable micronutrient for animals and plants. It is a component of chlorophyll and heme. It is also an important component of some enzymes and plays an essential role in the process of biological redox. A certain extent of iron is beneficial to the life activities of aquatic animals. However, with the development of the economy, too much excess iron is enriched in water. The excessive iron is poisonous and can cause poisoning or even death of aquatic plants and animals.

Industrial production waste liquid contains ferric ion. Iron exposure to the environment mainly occurs through mining, manufacturing units, and municipal or industrial wastewater. Iron pollution also occurs in response to corrosion of pipes and water supply from groundwater systems and from the atmosphere via rainwater [3]. When the same metals such as Fe accumulate at levels greater than the threshold level they become toxic. This induces the generation of reactive nitrogen and oxygen species (RNS; ROS), which results in the peroxidation of lipids in the plasma membrane [3]. In addition, iron packaging materials and iron products can also form waste ferric ion. These ferric ions cause water and soil pollution; in particular, the large amount of ferric ion in water causes severe water pollution and brings great harm to aquatic organisms [2, 4, 5]. The toxic effects of heavy metals on aquatic animals such as prawn and grouper have been studied intensively, and the results showed that heavy metal pollution may be further enriched between organisms through food chains [6–10], which in turn may threaten the survival of human. The accumulation of heavy metals in the human body leads to severe injury to various organs, specifically the respiratory, nervous, and reproductive systems and digestive tract [11–14]. Excess iron accumulation in tissue triggers iron-dependent oxidative stress [15]. Iron overload in the skeletal muscle not only negatively affects muscle contractility but also might impact its endocrine function, thus possibly affecting the clinical outcome of diseases, including neurodegenerative diseases [15]. Therefore, it is extremely important to effectively monitor water pollution and prevent the harmful impacts on biological health.

Planarian* Dugesia japonica* are flatworms (phylum Platyhelminthes) found in freshwater. They are widely distributed in China and have strong regeneration abilities. For over 200 years, planarians have been a classical model for studies on tissue regeneration [16, 17]. The body length of the planarian is generally 5-15 mm. They are soft, flat leaf-like, and bilateral symmetrical body shape. The head is triangular and the two eye-spots are used to detect the intensity of light. In addition, planarian is hermaphroditic; it can reproduce sexually or asexually. Previous studies showed that planarian is able to monitor water pollution [18, 19] and is sensitive to many pollutants [20–22]. The toxic effects of metal ions such as Cu^2+^ and Pb^2+^ and cadmium and other factors such as herbicides on planarian have been studied previously [15, 23–27]. Zhang Xiufang and Temenouga Guecheva confirmed that Cu^2+^ has a direct toxic effect on planarian, and planarian plays an important role in the biological monitoring of heavy metal copper ions [28, 29]. But there are few reports on the toxicity of iron ions to planarian.

In the present study, we focus on the effects of Fe^3+^ on the death and regeneration of* Dugesia japonica *under different temperatures; the results are useful for iron monitoring by planarian.

## 2. Materials and Methods

### 2.1. Experimental Materials

Planarian* Dugesia* ZB-1, a clonal propagation line of the* Dugesia japonica*, is preserved in our laboratory. FeCl_3_*∙*6H_2_O is produced by Tianjin Kaitong Chemical Reagent Co., Ltd.

### 2.2. Experimental Methods

#### 2.2.1. Preparation of Fe^3+^

10 g of analytically pure FeCl_3_*∙*6H_2_O crystals was dissolved in 100 mL distilled water to make 100 g/l iron solution. When used, dilute it to the exact concentration.

#### 2.2.2. Effect of Fe^3+^ on the Survival of Planarian

From the 100 g/l stock solution, four concentrations 40, 50, 60, and 70 mg/l of Fe^3+^ were made. Each concentration had 3 replicates. 10 planarians were placed in each concentration. At the same time, the four groups of planarian were cultured in 3 different constant temperature incubators: 15°C, 20°C, and 25°C. The culture medium was changed every day. The body changes of the planarian were observed at different time intervals.

#### 2.2.3. Effects of Fe^3+^ on the Regeneration of Planarian

Fe^3+^ was prepared in 100 g/l stock solution and then diluted to 10, 15, 20, and 30 mg/l solution. Each concentration had 3 replicates. 10 planarians were cut into three sections as head, trunk and tail and then placed in these four different concentrations. The four groups of regeneration concentrations were cultured at 15°C, 20°C, and 25°C. At the same time, set a cup of the same number of planarians in tap water at each temperature as control, and change the culture solution every day. The regeneration of the planarian was observed at different time intervals and repeated three times.

### 2.3. Data Processing

Data was performed by Graphpad Prism5 software, and statistical analysis was performed by Excel software and SPSS software.

## 3. Results

### 3.1. Effects of High Concentration of Fe^3+^ on the Survival of Planarian

#### 3.1.1. The Poisoning Form of Planarian

**Figure 1:**
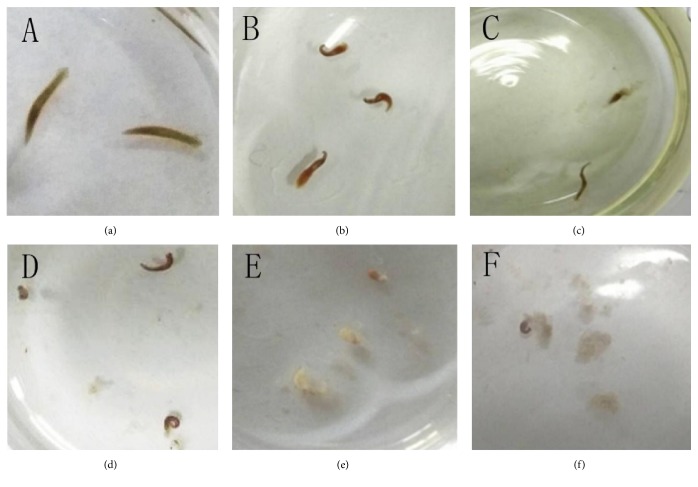
The poisoning pattern of the planarian on Fe^3+^. (a) The normal form of the planarian in clear water, naturally stretched; (b) the initial poisoning form of the planarian, the body is distorted, showing an “S” shape; (c-d) the head of the planarian is more sensitive, and the head disintegrates after the body is distorted; (e-f) as time goes by, the whole body of the planarian is completely decomposed, showing a diffuse, flocculent shape.

There were some differences in the toxic morphology of planarian in response to the various heavy metal ions. Planarians treated with different concentrations of Fe^3+^ showed that, in the early stage of treatments, the body of the planarian deformed and distorted, and the body seemed like “S” (Figure 1(b)). The effect was similar to these when the planarian was treated by copper and phenol [30]. After longer treatment the body state of the planarian in the lower concentration of Fe^3+^ returned to normal. However, the planarian shrank into a mass and stopped growing in the higher concentration of Fe^3+^. Additionally, the body of planarian was disintegrated (Figures 1(c) and 1(d)). Eventually, the whole body of the planarian was decomposed into white flocculence (Figures 1(e) and 1(f)), which indicated that long incubation time of high concentration of Fe^3+^ was able to make planarian die.

#### 3.1.2. Mortality Changes in Different Concentrations

**Figure 2:**
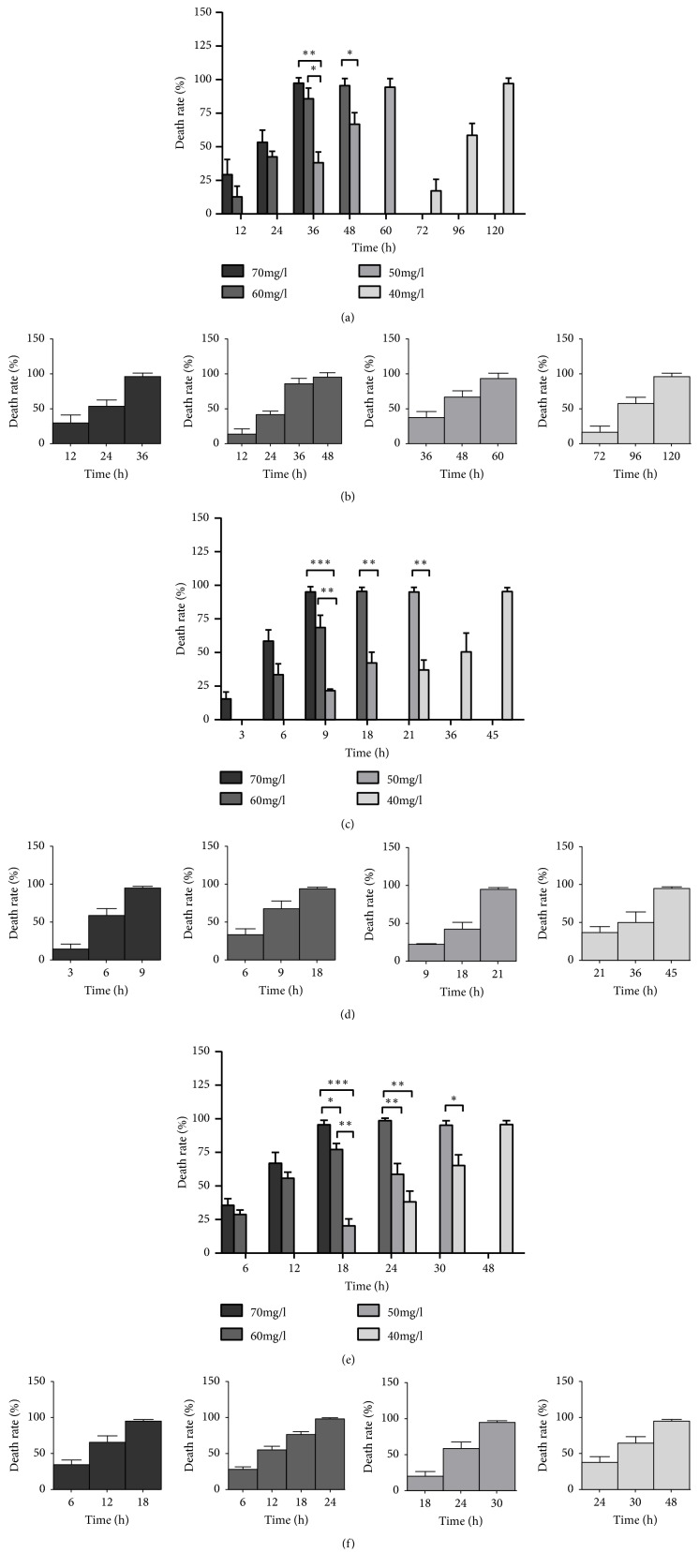
Effect of different temperatures and concentrations on the survival of planarian. (a-b) At 15°C, after treatment with different Fe^3+^ concentrations, the variation trend of the worm mortality was changed with time; (b) shows the change of mortality corresponding to each concentration; (c-d) similarly, it shows the distribution of the change in the mortality of the planarian after treatment at different concentrations of Fe^3+^ at 20°C; (e-f) changes in the mortality of the worms at different treatment concentrations at 25°C. Note: *∗∗∗* indicates P < 0.001, *∗∗* indicates P < 0.01 with extremely significant difference, and *∗* indicates P < 0.05 with significant difference.

At the same temperature, with the increase of Fe^3+^ concentration, the mortality of planarian increased gradually. At 15°C, all the planarian died after treatment for 36 h in 70 mg/l, 48 h in 60 mg/l, 60 h in 50 mg/l, and 120 h in 40 mg/l (Figures 2(a) and 2(b)). At 20°C and 25°C, there were obvious regularities between different concentrations; that is, the higher the concentration, the shorter the time to reach 100% mortality. At 20°C, all the planarian died after treatment for 9 h in 70 mg/l, 18 h in 60 mg/l, 21 h in 50 mg/l, and 45 h in 40 mg/l; at 25°C, all the planarian died after treatment for 18 h in 70 mg/l, 24 h in 60 mg/l, 30 h in 50 mg/l, and 48 h in 40 mg/l (Figures 2(c)–2(f)). The results indicated that the higher the concentration is, the faster the planarian die which revealed toxic effects of Fe^3+^ on planarian.

#### 3.1.3. Mortality Changes at Different Temperatures

Under the same Fe^3+^ concentration, there were also obvious regularities between different temperatures. Under 70 mg/l Fe^3+^, at 15°C, all planarian died after incubation for 36 h; at 25°C, all planarian died for 18 h, and at 20°C, all planarian died for 9 h (Figure 2). The differences were obvious. Similarly, when the concentrations were 60, 50, and 40 mg/l, from Figures 2, we could observe that the death rate was the highest at 20°C, but the lowest at 15°C, and the differences were significant. These mean that low temperature could slow down the effect of Fe^3+^ on the planarian toxicity, and at a suitable temperature, the toxic effects of Fe^3+^ on the planarian can be accelerated, which leads to the increased planarian mortality.

### 3.2. Effects of Nonlethal Concentrations of Fe^3+^ on the Regeneration of Planarian

It was found in the experiment that low concentration of Fe^3+^ did not cause the planarian death, and the change of poison status is not obvious. However, lower concentrations of Fe^3+^ can affect the regeneration process of the planarian.

#### 3.2.1. Changes in Regeneration Rate of Different Concentrations at the Same Temperature

**Figure 3:**
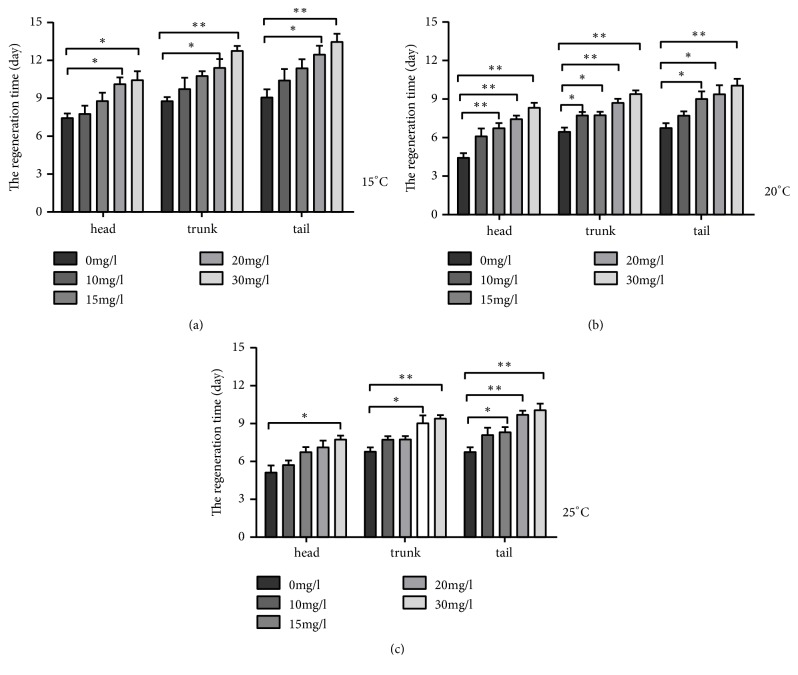
Effect of Fe^3+^ on the regeneration of planarian at different temperatures. (a) Contrast histogram of the number of days after regeneration of the head, trunk, and tail of the planarian at 15°C, under different treatment concentrations and water treatment; (b) similarly, at 20°C, contrast histogram of the number of days after the regeneration of the planarian; (c) similarly, the comparison histogram of the number of days of the regeneration of the planarian at 25°C. Note: *∗∗∗* indicates P < 0.001, *∗∗* indicates P < 0.01 with extremely significant difference, and *∗* indicates P < 0.05 with significant difference.

At the same temperature, as the iron concentration increased, the regeneration rate also became slow. At 15°C, 30 mg/l, the time when all three parts of the planarian regenerated completely is 10-14 days, which is a larger difference than the control group, which is 7 to 8 days; at 20 mg/l, the time when all three parts of the planarian regenerated completely is 10-12 days, also having a large difference compared with the control group (Figure 3(a)). Similarly, at 20°C and 25°C, with the increase of Fe^3+^ concentration, the regeneration completion times were correspondingly longer, and the differences between the experiment groups and the control group were significant (Figures 3(b) and 3(c)). The higher the concentration, the more significant the difference. According to a comprehensive comparison of the significant differences in different concentrations of the three groups of temperatures in Table 3, there was a significant difference between the different experiment groups and the control group. The different regeneration rate of the planarian between the treatment concentrations of each group indicates that the lower concentration of Fe^3+^ also affects the physiology of the planarian. The present study was similar to that of the published results, where the higher toxic concentration, the lower planarian regenerative capacity [15, 30].

#### 3.2.2. Changes in Regeneration Rate of Different Temperatures at the Same Concentration

At the same concentration, the results showed that the regeneration rate of the planarian was the slowest at 15°C and the fastest at 20°C (Figure 3). When the concentration was 30 mg/l, the average regeneration time of the head, trunk, and tail parts at 15°C was about 10-14 days (Figure 3(a)), while the regeneration time was not more than 9 days at 20°C (Figure 3(b)). The difference is obvious, and the same is true at the concentrations of 20 mg/l, 15 mg/l, and 10 mg/l. It showed that Fe^3+^ had a low toxic effect on the planarian in a low temperature, and at a suitable temperature, the regeneration rate of the planarian could be significantly suppressed. According to the results displayed in Table 1, there was a significant difference in the regeneration rate between 15°C and 20°C and between 15°C and 25°C, but there is no significant difference between 20°C and 25°C. The speeds of the planarian regeneration were not much different, and it is estimated that the suitable regeneration temperature of planarian would be between 20°C and 25°C.

## 4. Discussion

### 4.1. Effect of Fe^3+^ on the Survival of Planarian at Different Temperatures

Horvat T described that the herbicide might cause damage to the outer mucosa and other tissues of planarian and cause DNA damage [25]. Pagan OR reported that dimethyl sulfoxide (DMSO) can affect various aspects of the toxicity and behavioral effects in the planarian, revealing restricted levels of drug use and the effects on aquatic organisms [31, 32], and Guecheva Temenouga N [23] studied the effects of copper on the stressor protein response and catalase activity. In the study of Ofoegbu PU [33], the work showed that the* S. mediterranea* is sensitive to TBT, and TBT can influence the planarian survival, locomotion, head regeneration, and DNA damage. In the present study, iron ions are used to investigate the poison effect to planarian. Iron ions are a kind of trace elements necessary for animals and plants, and they are also a kind of water pollutants.

The study compared the effects of Fe^3+^ on the mortality of the planarian at different temperatures for different time periods. The mortality of the control group was 0. At the same temperature and different concentrations, with the increase of the concentration of Fe^3+^, the mortality of the planarian was distinct. The higher the concentration of Fe^3+^, the higher the mortality. At 15°C, the planarian all died in the 70 mg/l concentration of Fe^3+^ solution for about 36 h (Figures 2(a) and 2(b)). When the concentration was decreased, the time to reach 100% mortality is also extended, but it still caused death damage to the planarian body. Similarly, at 20°C and 25°C, the mortality rate was increased because of the increased concentration. At the same concentration and different temperatures, it can be seen from Figure 2 that, at the lowering temperature of 15°C, the death rate of the planarian is slower than other temperatures. At 20°C, the rate of death of the planarian was the fastest. This may be caused by the high activity of the related enzymes and the fastest metabolism at 20°C, which may accelerate the toxic effects of Fe^3+^ on the planarian. The slow metabolism at 15°C may weaken the toxic effect of Fe^3+^. At 25°C, the temperature-induced stress on the planarian was not obvious, and the effect of the worm's metabolism on the toxic effect was slightly smaller than that at 20°C. That is, the speed of the planarian death at the same concentration at 20°C is the fastest and at 25°C and 15°C is the slowest.

### 4.2. Effects of Fe^3+^ on the Regeneration of Planarian at Different Temperatures

According to the analysis, at the same temperature and different concentrations, with the increase of Fe^3+^ concentration, the regeneration rate of the head, trunk, tail, and adult of the planarian decreased, and the regeneration completion time was prolonged. At the same concentration and different temperature, the regeneration rate of the planarian at 20°C is slightly faster than that of the other temperature conditions, but it is not much different from that at 25°C (Table 1). The statistical analysis by SPSS software showed that there was a significant difference between 15°C and 20°C and between 15°C and 25°C, but there was no significant difference between 20°C and 25°C, which indicated that low temperature has a significant impact on the rate of regeneration of the planarian, and it was estimated that the optimum regeneration temperature of the planarian should be between 20°C and 25°C. Temperature can affect many biology aspects of planarian* Schmidtea mediterranea* [34]. As to planarian* Dugesia japonica*, in the previous study, the results showed that the optimal regeneration temperature of the eye point is 22°C [35, 36], and the optimal regeneration temperature of the planarian was 22°C [37], which was consistent with the present study. But there are differences about planarian* Schmidtea mediterranea*; it was reported that, at 19°C, the appearance of the eyes from the trunk was confirmed at five days after amputation; at 26°C and 28°C, the eyes were observed at three days after amputation[28]. However, the optimal living conditions of the planarian are 18°C-20°C; whether it is easier to regenerate for lower creatures under adversity condition, further research is needed. The regeneration rate was the slowest at 15°C, which may be because of the low activity of related enzymes in the planarian. In addition, Table 3 showed the differences between the different treatment concentrations. There were significant differences between groups which further showed that Fe^3+^ had strong toxic effects on the regeneration of planarian. In addition, it was found that there was decomposition of the head of the 30 mg/l Fe^3+^ treated planarian at 20°C and 25°C, indicating that the head of the planarian is less resistant to toxicity, so the survival rate was lower than that of the trunk and the tail parts, but the specific reasons still need further research. In this analysis, the time of regeneration of the head was the fastest. Results showed that there were significant differences in regeneration rates between different body parts (Table 2), but in natural conditions, they already showed the regeneration differences between different body parts of the planarian. So, whether Fe^3+^ affects the regeneration rate and how does it work still need further study.

## Figures and Tables

**Table 1:** Comparison of significant differences among the three groups at different temperatures.

Dependent Variable: Regeneration days							
	(I) temperature	(J) temperature	Mean Difference (I-J)	Std. Error	Sig.	95% Confidence Interval	
						Lower Bound	Upper Bound
LSD	15°C	20°C	2.56^*∗*^	.182	.000	2.19	2.92
		25°C	2.56^*∗*^	.182	.000	2.19	2.92
	20°C	15°C	-2.56^*∗*^	.182	.000	-2.92	-2.19
		25°C	.00	.182	1.000	-.36	.36
	25°C	15°C	-2.56^*∗*^	.182	.000	-2.92	-2.19
		20°C	.00	.182	1.000	-.36	.36

Based on observed means.

The error term is Mean Square (Error) = 0.748.

*∗* The mean difference is significant at the 0.05 level.

**Table 2:** Comparison of regeneration days in planarian head, trunk and tail fragments.

Dependent Variable: Regeneration days							
	(I)Part	(J) Part	Mean Difference (I-J)	Std. Error	Sig.	95% Confidence Interval	
						Lower Bound	Upper Bound
LSD	Head	trunk	-1.62^*∗*^	.182	.000	-1.98	-1.26
		Tail	-2.20^*∗*^	.182	.000	-2.56	-1.84
	trunk	Head	1.62^*∗*^	.182	.000	1.26	1.98
		Tail	-.58^*∗*^	.182	.002	-.94	-.22
	Tail	Head	2.20^*∗*^	.182	.000	1.84	2.56
		trunk	.58^*∗*^	.182	.002	.22	.94

Based on observed means.

The error term is Mean Square (Error) =0.748.

*∗* The mean difference is significant at the 0.05 level.

**Table 3:** Comparison of significant differences among the different concentrations of iron.

Dependent Variable: Regeneration days							
	(I)concentration	(J) concentration	Mean Difference (I-J)	Std. Error	Sig.	95% Confidence Interval	
						Lower Bound	Upper Bound
LSD	0mg/l	10mg/l	-1.07^*∗*^	.235	.000	-1.54	-.61
		15mg/l	-1.78^*∗*^	.235	.000	-2.25	-1.31
		20mg/l	-2.67^*∗*^	.235	.000	-3.13	-2.20
		30mg/l	-3.37^*∗*^	.235	.000	-3.84	-2.90
	10mg/l	0mg/l	1.07^*∗*^	.235	.000	.61	1.54
		15mg/l	-.70^*∗*^	.235	.004	-1.17	-.24
		20mg/l	-1.59^*∗*^	.235	.000	-2.06	-1.12
		30mg/l	-2.30^*∗*^	.235	.000	-2.76	-1.83
	15mg/l	0mg/l	1.78^*∗*^	.235	.000	1.31	2.25
		10mg/l	.70^*∗*^	.235	.004	.24	1.17
		20mg/l	-.89^*∗*^	.235	.000	-1.36	-.42
		30mg/l	-1.59^*∗*^	.235	.000	-2.06	-1.12
	20mg/l	0mg/l	2.67^*∗*^	.235	.000	2.20	3.13
		10mg/l	1.59^*∗*^	.235	.000	1.12	2.06
		15mg/l	.89^*∗*^	.235	.000	.42	1.36
		30mg/l	-.70^*∗*^	.235	.004	-1.17	-.24
	30mg/l	0mg/l	3.37^*∗*^	.235	.000	2.90	3.84
		10mg/l	2.30^*∗*^	.235	.000	1.83	2.76
		15mg/l	1.59^*∗*^	.235	.000	1.12	2.06
		20mg/l	.70^*∗*^	.235	.004	.24	1.17

Based on observed means.

The error term is Mean Square (Error) =0.748.

*∗* The mean difference is significant at the 0.05 level.

## Data Availability

The data used to support the findings of this study are available from the corresponding author upon request.
